# Integrative analysis provides multi‐omics evidence for the pathogenesis of placenta percreta

**DOI:** 10.1111/jcmm.15973

**Published:** 2020-10-21

**Authors:** Qingyuan Jiang, Lei Dai, Na Chen, Junshu Li, Yan Gao, Jing Zhao, Li Ding, Chengbin Xie, Xiaolian Yi, Hongxin Deng, Xiaodong Wang

**Affiliations:** ^1^ Department of Obstetrics and Gynecology West China Second University Hospital of Sichuan University and Key Laboratory of Birth Defects and Related Diseases of Women and Children (Sichuan University) Ministry of Education Chengdu China; ^2^ Department of Obstetrics Sichuan Provincial Hospital for Women and Children Chengdu China; ^3^ State Key Laboratory of Biotherapy and Cancer Center West China Hospital Sichuan University Chengdu China; ^4^ Imaging Center Sichuan Provincial Hospital for Women and Children Chengdu China; ^5^ Department of Laboratory Medicine Sichuan Provincial Hospital for Women and Children Chengdu China; ^6^ Pathology Department Sichuan Provincial Hospital for Women and Children Chengdu China

**Keywords:** lncRNA, miRNA, pernicious placenta previa, placenta percreta, *Wnt5A*

## Abstract

Pernicious placenta previa with placenta percreta (PP) is a catastrophic condition during pregnancy. However, the underlying pathogenesis remains unclear. In the present study, the placental tissues of normal cases and PP tissues of pernicious placenta previa cases were collected to determine the expression profile of protein‐coding genes, miRNAs, and lncRNAs through sequencing. Weighted gene co‐expression network analysis (WGCNA), accompanied by miRNA target prediction and correlation analysis, were employed to select potential hub protein‐coding genes and lncRNAs. The expression levels of selected protein‐coding genes, *Wnt5A* and *MAPK13*, were determined by quantitative PCR and immunohistochemical staining, and lncRNA *PTCHD1‐AS* and *PAPPA‐AS1* expression levels were determined by quantitative PCR and fluorescence in situ hybridization. The results indicated that 790 protein‐coding genes, 382 miRNAs, and 541 lncRNAs were dysregulated in PP tissues, compared with normal tissues. WGCNA identified coding genes in the module (ME) black and ME turquoise modules that may be involved in the pathogenesis of PP. The selected potential hub protein‐coding genes, *Wnt5A* and *MAPK13*, were down‐regulated in PP tissues, and their expression levels were positively correlated with the expression levels of *PTCHD1‐AS* and *PAPPA‐AS1*. Further analysis demonstrated that *PTCHD1‐AS* and *PAPPA‐AS1* regulated Wnt5A and MAPK13 expression by interacting with specific miRNAs. Collectively, our results provided multi‐omics data to better understand the pathogenesis of PP and help identify predictive biomarkers and therapeutic targets for PP.

## 1 INTRODUCTION

Placenta accreta is an abnormal placental attachment caused by the invasion of placental villi into the myometrium. According to the depth of placental invasion into the myometrium and degree of infiltration into the organs adjacent to the uterus, abnormal placental attachment can occur as follows: (a) placenta accreta: placenta invades the superficial myometrium of the uterus; (b) placenta increta: placenta invades the deep myometrium of the uterus; and (c) placenta percreta (PP): placenta penetrates the uterine wall and reaches the serous layer of the uterus and even invades the organs adjacent to the uterus.^1^ The main risk factors for placenta accreta include placenta previa, history of previous caesarean section, history of intrauterine surgery, pregnancy by in vitro fertilization‐embryo transfer, advanced age, and history of uterine perforation.^2^, ^3^, ^4^ In 1993, Chattopadhyay et al investigated the relationship between placenta previa and placenta accreta and previous caesarean section and put forward the concept of pernicious placenta previa for the first time.^5^ Pernicious placenta previa with placenta accreta is an important cause of perinatal hysterectomy, premature delivery, and perinatal death. The hysterectomy rate of patients is as high as 66%, often accompanied by bladder and ureteral injury, and maternal mortality caused by massive haemorrhage is as high as 7%.^6^, ^7^, ^8^ The process of placenta accreta development is complex, and its pathogenesis is gaining more attention. Loss of decidua,^9^, ^10^ enhanced invasiveness of trophoblasts^11^, ^12^, ^13^, ^14^ and abnormal recasting of uterine spiral arteries are considered to be the three important pathophysiological bases that lead to placenta accrete by interacting with and influencing each other.^14^, ^15^, ^16^, ^17^ Recently, it has been shown that some important coding and non‐coding genes are closely related to placenta accreta, such as those that encode tumour necrosis factor‐related apoptosis‐inducing ligand‐receptor 2 (TRAIL‐R2), miR‐29 and miR‐519d.^18^, ^19^, ^20^ However, the regulatory mechanisms of the molecular networks related to placenta accreta remain unclear.

Long non‐coding RNAs (lncRNAs) are a class of transcripts longer than 200 base pairs that generally do not code for proteins. LncRNAs exert diverse roles in cellular and biological processes via the regulation of gene expression and chromatin dynamics.^21^ Recently, lncRNAs have been shown to contribute to the pathogenesis of various diseases, including cancer,^22^, ^23^, ^24^ cardiovascular disease^25^ and nervous system diseases.^26^ The deregulation of lncRNAs IGF2/H19, MEG3, SPRY4‐IT1, HOTAIR, MALAT1, FLT1P1 and CEACAMP8 in placental trophoblasts is involved in the pathogenesis of preeclampsia.^27^ Among them, lncRNA MALAT1 may be involved in the pathogenesis of preeclampsia via the regulation of the proliferation, cell cycle, apoptosis, migration and invasion of trophoblast cells.^28^ Thus, we speculated that lncRNAs may also be deregulated in the placental tissues of pregnant women with placenta accreta spectrum (PAS) and play a crucial role in the pathogenesis of PAS.

Therefore, in this study, the different pathological features of normal placental tissue and penetrating placental tissue were compared. Additionally, the expression profiles of coding genes, lncRNAs and miRNAs in these two types of placental tissue were compared and analysed using multi‐omics. Weight gene co‐expression network analysis (WGCNA), accompanied by miRNA target prediction and correlation analysis, was employed to select potential hub coding genes and lncRNAs. The expression of the selected coding genes, *Wnt5A* and *MAPK13*, was determined by quantitative PCR (qPCR) and immunohistochemical staining and that of lncRNAs PTCHD1‐AS and PAPPA‐AS1 was determined by qPCR and fluorescence in situ hybridization (FISH) staining. The results have demonstrated the expression profiles of lncRNAs, miRNAs and coding genes in PP and provided multi‐omics evidence to explain the pathogenesis of placenta accreta.

## 2 MATERIALS AND METHODS

### 2.1 Clinical samples

All clinical samples were obtained from Sichuan Provincial Hospital for Women and Children, and the experiments were approved by the Medical Ethics Committee of the hospital (Approval Number: 20191125‐38). All pregnant women included in the study were enrolled in accordance with surgical indications (Table 1), and clinical samples were collected receiving participants' informed consent. Before surgery, the participants were diagnosed using magnetic resonance imaging and ultrasonication at Sichuan Provincial Hospital for Women and Children. Criteria for the experimental group were as follows: (a) all the patients were diagnosed with pernicious placenta previa with PP; (b) all patients underwent hysterectomy; (c) no infection and no internal and surgical complications; (d) all cases were excluded from other pregnancy complications. In the experimental group, fresh sterile penetrating part of placenta from maternal surface was selected as tissue samples after hysterectomy was performed. While normal placenta tissue from maternal surface was selected as the samples in the control group during caesarean section without obstetric complications. The placenta tissue samples of normal pregnancy and PP are all taken from the same site. Eight placenta tissue samples (including 4 placenta tissues samples of normal pregnancy and 4 PP) are used for RNA sequencing, and 12 placenta tissue samples (including 6 placenta tissues samples of normal pregnancy and 6 PP) are used for qPCR, IHC staining and FISH staining.

**TABLE 1:** Clinical characteristics of pregnant woman

Type	Age	Weight (kg)	Gestational weeks	Gestational times	Times of parturition	Times of caesarean	Indication of operation	Other complications
Normal‐1	34	65	39.3	4	2	1	Scarred uterus	None
Normal‐2	25	80	40.1	1	1	0	Foetal macrosomia	None
Normal‐3	29	66	39.2	2	1	0	Breech presentation	None
Normal‐4	27	64	39.0	1	1	0	Cord around neck	None
Normal‐5	32	54	40.0	2	2	0	Foetal distress	None
Normal‐6	31	77	39.4	1	1	0	Foetal macrosomia	None
PP‐1	24	60	34.5	7	3	2	Pernicious placenta previa	None
PP‐2	28	76	39.1	5	2	1	Pernicious placenta previa	None
PP‐3	34	70	35.2	3	3	2	Pernicious placenta previa	None
PP‐4	29	60	35.2	4	2	1	Pernicious placenta previa	None
PP‐5	34	71	35.0	3	2	1	Pernicious placenta previa	None
PP‐6	39	60	35.2	3	3	2	Pernicious placenta previa	None

Abbreviation: PP, placenta percreta.

### 2.2 RNA extraction and sequencing

Total RNA samples were extracted following the instruction of Trizol kit (Invitrogen) and their quality and quantity were determined as follows. The samples were first qualified using 1% agarose gel electrophoresis to detect possible contamination and degradation. RNA purity and concentration were then determined using a NanoPhotometer^®^ spectrophotometer (Implen, Munich, Germany). Finally, RNA integrity and quantity were measured using the RNA Nano 6000 Assay Kit and the Bioanalyzer 2100 system (Agilent, Santa Clara, CA, USA). RNA sequencing was performed by Chengdu Basebiotech Co., Ltd (Chengdu, China) as follow: a total of 1 μg of RNA per sample was used as the input material for RNA sample preparations. Sequencing libraries were generated using NEBNext^®^ UltraTM RNA Library Prep Kit for Illumina^®^ (NEB, Ipswich, MA, USA) following the manufacturer's recommendations, and index codes were added to attribute sequences to each sample. Briefly, mRNA was purified from total RNA using poly T oligo‐coupled magnetic beads. Fragmentation was performed using divalent cations under elevated temperature in NEBNext First Strand Synthesis Reaction Buffer (5×). First‐strand cDNA was synthesized using random hexamer primers and M‐MuLV Reverse Transcriptase (RNase H‐). Second‐strand cDNA synthesis was subsequently performed using DNA polymerase I and RNase H. Remaining overhangs were converted into blunt ends via exonuclease and polymerase activities. After adenylation of the 3′ ends of the DNA fragments, an NEBNext Adapter, with a hairpin loop, structure was ligated to prepare for hybridization. To preferentially select cDNA fragments of 250‐300 bp, library fragments were purified using the AMPure XP system (Beckman Coulter, Beverly, MA, USA). Prior to PCR, size‐selected, adapter‐ligated cDNA samples were then incubated with 3 μL of USER Enzyme (NEB) at 37°C for 15 minutes, followed by 5 minutes at 95°C. PCR was performed using Phusion High‐Fidelity DNA polymerase, universal PCR primers, and Index (X) Primers. Finally, PCR products were purified using the AMPure XP system and library quality was assessed on an Agilent Bioanalyzer 2100 system. The clustering of the index‐coded samples was performed on a cBot‐Cluster Generation System using TruSeq PE Cluster Kit v3‐cBot‐HS (Illumia) according to the manufacturer's instructions. After cluster generation, the library preparations were sequenced on an Illumina Novaseq platform and 150 bp paired‐end reads were generated. Significantly differentially expressed lncRNAs, miRNAs, and protein‐coding genes were screened based on absolute value of log2 (fold change) ≥ 1, at a *P* value <0.05.

### 2.3 Weighted gene co‐expression network analysis

Using standard weighted gene co‐expression network analysis (WGCNA) procedures, a network was constructed using the WGCNA package in R (https://horvath.genetics.ucla.edu/html/CoexpressionNetwork/Rpackages/WGCNA/) and data were visualized using the Cytoscape software. The flashCluster package in R software was first used to analyse the samples and identify abnormal values. The WGCNA adjacency function was then used to create an adjacency matrix and calculate Pearson's correlations, to determine the consistency of gene expression levels between each gene pair.^29^ Next, we used the topological overlap matrix (TOM) similarity function to transform the matrix into a TOM. Finally, co‐expression modules were constructed using the WGCNA algorithm, and the gene information of each module was extracted.

### 2.4 Kyoto Encyclopedia of Genes and Genomes pathway analysis

The Kyoto Encyclopedia of Genes and Genomes (KEGG) is a set of databases that provides a comprehensive understanding of biological systems. It can be used to analyse biological pathways and genes related to diseases and drugs. KEGG pathway datasets were downloaded from http://www.genome.jp/kegg/pathway.html. KEGG pathway enrichment was analysed using the ‘ggplot2’ R package and the online biological tool, KOBAS 3.0 (http://kobas.cbi.pku.edu.cn). *P* < 0.05 was considered to indicate statistical significance.

### 2.5 Target prediction

miRNA target binding information was obtained from the mirwalk2 website (http://zmf.umm.uni‐heidelberg.de/apps/zmf/mirwalk2/). For lncRNA and protein‐coding genes, the default target relationship in mirwalk2 was used.

### 2.6 Predicting lncRNAs of sponge regulatory network and co‐expression module genes

We obtained a list of genes from the clinical trait‐related co‐expression module. We focused on genes that showed differential expression between normal and diseased tissue. We devised a computational strategy to identify candidate lncRNA‐gene pairs based on sponge regulatory network. Firstly, for each lncRNA‐driver gene pair, we estimated the significance of shared miRNAs with the same seeds (*P*‐value of one‐tailed Fisher's exact test) and the significance of expression correlation across all samples. We then computed a combined *P*‐value by converting the *P*‐values of these two tests, P1 and P2, using the sum of logs method (also called Fisher's method) with the metap package. The candidate lncRNA‐RNA‐driver gene pairs met the criterion that the adjusted combined *P*‐value was no larger than a threshold of 5% (ie false discovery rate, *r* < 0.05). Secondly, we selected lncRNA‐protein‐coding gene pairs that shared at least ten different miRNAs. Finally, we selected RNA‐driver gene pairs that showed at least a moderate positive correlation of their expression levels (*r* > 0.25). All lncRNAs, genes and miRNAs were filtered to show differential expression at a cutoff *P*‐value < 0.05. The top 20 miRNAs ranked by *P*‐value were plotted. All of the analysis were conducted according to method report by previous study,^30^ with in‐house R scripts. ceRNA networks were visualized with Cytoscapesoftwares.

### 2.7 Venn analysis

To complete the Venn map, we prepared a list of differential genes in each group. We then used the mapping website (http://bioinfogp.cnb.csic.es/tools/venny/index.html) to obtain a Venn graph.

### 2.8 Quantitative PCR

Total RNA was extracted from tissue samples using TRIzol (Life Technologies, Carlsbad, CA, USA). RNA samples were dissolved in RNase‐free water and quantified using a NanoDrop2000 instrument (Thermo Fisher Scientific, Waltham, MA, USA). A PrimeScript^TM^ RT reagent kit with gDNA Eraser (Real Time Perfect; TaKaRa, Kusatsu, Japan) was used to generate cDNA from 1 μg of RNA, according to the manufacturer's instructions. Next, SYBR Premix Ex Taq^TM^ (TliRNaseH Plus II; TaKaRa) was used for highly specific amplification, using the following parameters: 1 cycle of 30 seconds at 95°C, followed by 42 cycles of 5 seconds at 95°C and 30 seconds at 58°C. Melting curve analyses were performed to verify primer specificities. All of the PCR reactions and melting curve analyses were performed on LightCycler 96 platform (Roche). RNA expression levels were calculated using the 2^−ΔΔC^ *^t^* method. The primers for miRNA detection and U6 detection were purchased from Ribo Bio (Guangzhou, China), and the sequences were not supplied according the rules of the company. The sequences of lncRNA and coding genes primers used for qPCR were listed as follow:

**Table:** 

Genes (accession number in NCBI database)	Forward primer (5′‐3′)	Reverse primer (5′‐3′)
FAM225A (NR_024366.1)	GCTGCTGAGAGTGTCTAAGGA	TGTGCGACGGTGCTGAAT
LINC00941 (NR_040245.1)	AACAGACCAGACCAGAAGAGT	GGCAGCAAGAATGAGAGTTGA
LINC00994 (NR_033978.1)	AGTCTTCCTTGCCTGCTCTTAT	ATACTGCTCCGTGTGGTCTC
LINC00501 (NR_047465.1)	CATTCTTCCTTCTGTGGCTTGA	ATCCTGGCTAACACGGTGAA
PTCHD1‐AS (NR_073010.2)	AGTCTCTGCAACGGTCCTCT	ATGTTCCATCATGCTCCCAGG
PAPPA‐AS1 (NR_103711.1)	GGACTGTGCAGTGTGGTTCT	GTAGGGAGTGGGAGGGCTTA
BTNL9 (NM_001308245)2	CGCTTCCACTCCGACAACT	CGCTTCCACTCCGACAACT
MAGEA4 (NM_001011548.1)	CTGTCTCCTCCTCCTCTCCT	AGCCAACTCATCCACCTTGT
ARHGEF28 (NM_001080479.3)	TCTTCCTCCGTGCCTGTTG	TGACATCCTTCTCCTGCCTATT
NR4A3 (NM_006981.4)	CAACTACGAACTCAAGCCTTCC	GTGGTGGTGATGGTGATGGT
Wnt5A (NM_001256105.1)	GGTGGTCGCTAGGTATGA	TCGGAATTGATACTGGCATT
MAPK13 (NM_002754.5)	GCTCCTGGATGTCTTCAC	TTCAGTTCACAGTCCTCATT
GAPDH (NM_001256799.3)	TCAAGGCTGAGAACGGGAAG	TCGCCCCACTTGATTTTGGA

### 2.9 Immunohistochemical staining

Tissue samples from patients were fixed in 4% paraformaldehyde at room temperature (22‐25°C), embedded in paraffin, and cut into 5‐μm‐thick sections. The sections were then deparaffinized with xylene, rehydrated in a graded ethanol series, and washed in ultrapure water. They were autoclaved at 120°C for 5 minutes for antigen retrieval with citrate repair buffer (pH 6.0, Origene) and cooled at room temperature for 20 minutes. After antigen retrieval, sections were incubated in 3% H_2_O_2_ and 5% goat serum at room temperature for 15 minutes. They were then incubated with anti‐Wnt5a (ab229200; Abcam, Cambridge, UK, 1:100 dilution) or anti‐MAPK13 (ab236738; Abcam, Cambridge, MA, USA, 1:50 dilution) primary antibodies overnight at 4°C, followed by incubation with secondary antibodies (HRP kit, Cat. No. SP9001; Zsbio, Beijing, China) and HRP‐conjugated streptomycin solution (HRP kit, Cat. No. SP9001; Zsbio) for 30 minutes at 37°C, separately. Finally, the sections were stained with 3,3‐diaminobenzidine (Maixin, Fuzhou, China) and counterstained with haematoxylin.

### 2.10 Fluorescence in situ hybridization

Specific fluorescence in situ hybridization (FISH) probes labelled with Cy3 were designed and synthesized by RiboBio (Guangzhou, China). FISH procedures were performed according to the manufacturer's instructions of Fluorescent In Situ Hybridization Kit (RiboBio). Tissues were fixed with 4% paraformaldehyde at room temperature (22‐25°C); cut into 5‐μm‐thick sections; and hydrated in 100%, 85%, and 70% ethanol. After washing with PBS, tissue sections were digested with 0.1 g/mL pepsin at 37°C for 30 minutes, followed by successive dehydration in 70%, 85% and 100% alcohol. The sections were then hybridized with Cy3‐labelled probes at 83°C for 5 minutes and 42°C overnight. DAPI was used to counterstain the cell nuclei. A laser‐scanning microscope (A1, Nikon) was used to observe the subcellular distribution of lncRNAs in the tissue sections. NIS‐Elements C platform was used for image acquisition.

### 2.11 Statistical analysis

All data were analysed by a paired *t* test, using GraphPad Prism 5.0 (GraphPad software, Inc, San Diego, CA, USA). A *P*‐value < 0.05 was considered statistically significant. All experiments were performed on three or more independent occasions, and the data are presented as the mean ± standard error.

## 3 RESULTS

### 3.1 Clinical characteristics of the collected samples

**FIGURE 1:**
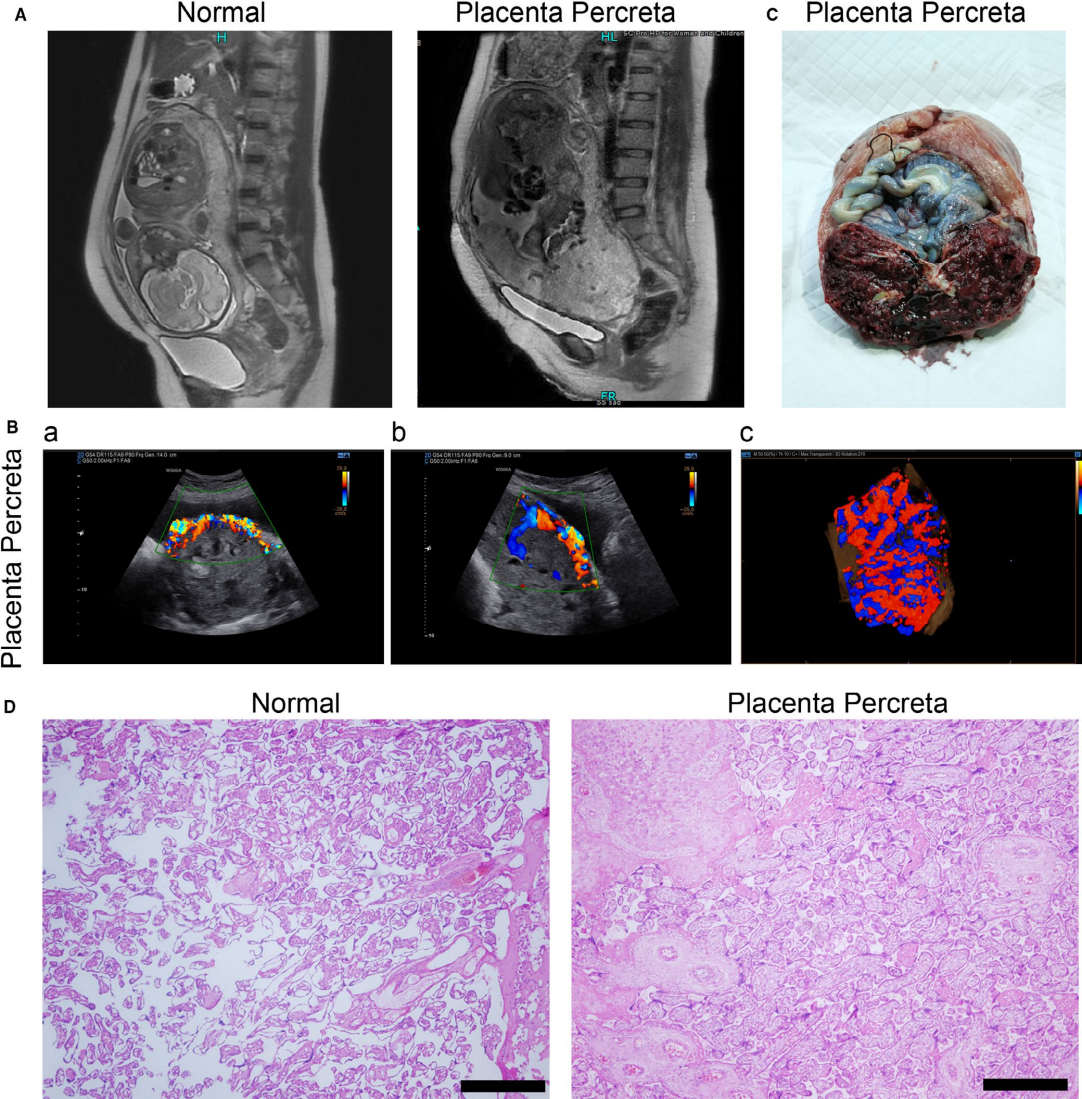
Clinical characteristics of collected samples. (A) Magnetic resonance imaging (MRI) and (B) ultrasonic examination of normal and placenta percreta groups. The transection (a), sagittal section (b) and three‐dimensional (c) flow diagram are presented. (C) White light image of tissue samples taken during surgery. (D) Haematoxylin and eosin staining of tissue samples taken during surgery. Scale bar = 200 μm

To investigate the pathogenesis of placenta accreta, placental tissues from pregnant women with normal placenta and those with pernicious placenta previa with PP who met the surgical indications were collected for further sequencing. The detailed clinical characteristics of the pregnant women included in the study are listed in Table 1. Magnetic resonance imaging (MRI) showed that the placenta was located in the normal position of the posterior wall of the uterus without adhesion implantation in the normal group (Figure 1A). In the PP group, the placenta completely covered the inner cervix, accompanied by abundant blood vessels in the cervix and the disappearance of normal inner cervix morphology (Figure 1A). The placenta above the inner mouth was 8 cm thicker in the PP group than in the normal group and the muscular layer of the lower part of the anterior uterine wall was very thin and adhered to the bladder (Figure 1A). Ultrasonic examination showed penetrating placenta implantation, the disappearance of the posterior placental gap at the lower incision of the anterior uterine wall, and rich comb‐like blood flow in the pernicious placenta previa group (Figure 1B). Operative uterine and placental specimens showed that the placenta completely covered the internal os of the cervix was implanted in the myometrium and penetrated the serous layer (Figure 1C). H&E staining results indicated that the myometrium was absent in the placental tissues of the percreta group, whereas the normal group had normal placental tissue (Figure 1D). These results clarified the pathological features of the placental tissues for sequencing analysis.

### 3.2 Different expression of coding genes, miRNAs and lncRNAs in PP

**FIGURE 2:**
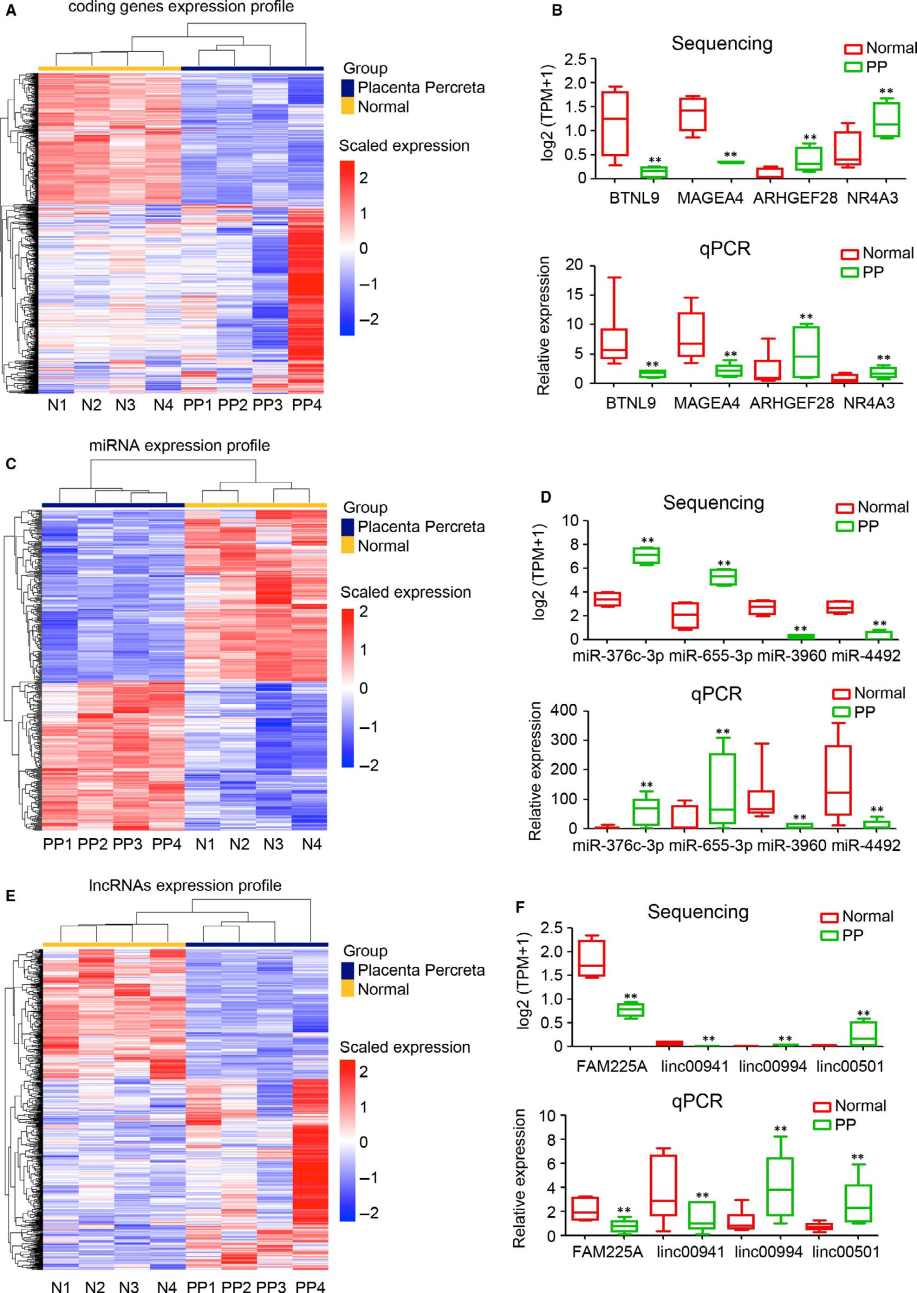
Expression profile of lncRNAs, miRNAs and mRNAs. A, Expression profile of protein‐coding mRNAs in the placental tissue of normal and placenta percreta (PP) groups. B, The expression levels of four randomly selected protein‐coding genes, *BTNL9*, *MAGEA4*, *ARHGEF28* and *NR4A3* in placental tissue of normal and PP groups, as determined by RNA sequencing and qPCR (n = 6 per group; **P < 0.01 compared with the normal group. GAPDH was used as a loading control.). C, Expression profile of miRNAs in placental tissue of normal and PP groups. D, The expression levels of four randomly selected miRNAs, *miR‐376c‐3p*, *655‐3p*, *3960* and *4492*, in placental tissue of normal and PP groups, as determined by RNA sequencing and qPCR (n = 6 per group; **P < 0.01 compared with the normal group. U6 was used as a loading control). E, Expression profile of lncRNAs in placental tissue of normal and PP groups. F, The expression levels of four randomly selected lncRNAs, *FAM225A*, *linc00941*, *linc00994* and *linc00501* in placental tissue of normal and PP groups, as determined by RNA sequencing and qPCR (n = 6 per group; **P < 0.01 compared with the normal group. GAPDH was used as a loading control.)

To determine the potential pathogenesis of PP, the expression profiles of genes (coding genes, miRNAs and lncRNAs) were determined by sequencing. A heatmap displays the differential expression of coding genes in the placental tissues of the placenta increta and normal groups (Figure 2A). The results showed that 469 coding genes were up‐regulated in the PP group, whereas 321 coding genes were down‐regulated, compared with the normal group (Table 2). To confirm the accuracy of sequencing, qPCR was employed to determine the expression of four randomly selected coding genes (*BTNL9*, *MAGEA4*, *ARHGEF28* and *NR4A3*). The expression trend determined by qPCR was consistent with the results of sequencing (Figure 2B). Kyoto Encyclopedia of Genes and Genomes analysis indicated that up‐regulated coding genes regulate the ErbB signalling pathway, bladder cancer and osteoclast differentiation (Figure S1A), whereas down‐regulated coding genes are involved in apoptosis—multiple species, cytokine‐cytokine receptor interaction and thiamine metabolism (Figure S1B). miRNA sequencing showed that 178 miRNAs were up‐regulated in the PP group, whereas 204 miRNAs were down‐regulated, compared with the normal group (Figure 2C; Table 2). The expression trends of miR‐376c‐3p, ‐655‐3p, ‐3960 and ‐4492 were consistent between the sequencing and qPCR results (Figure 2D). In total, 322 up‐regulated lncRNAs and 219 down‐regulated lncRNAs were identified in the PP group compared with the normal group (Figure 2E; Table 2). The qPCR results of four randomly selected lncRNAs (FAM225A, linc00941, linc00994 and linc00501) confirmed the accuracy of lncRNA sequencing (Figure 2F). These results displayed the expression profile of lncRNAs, miRNAs and coding genes in PP.

**TABLE 2:** Number of deregulated genes in placenta percreta

Genes	Up‐regulated	Down‐regulated
Coding RNAs	469	321
miRNAs	178	204
lncRNA	322	219

### 3.3 WGCNA of deregulated coding genes

**FIGURE 3:**
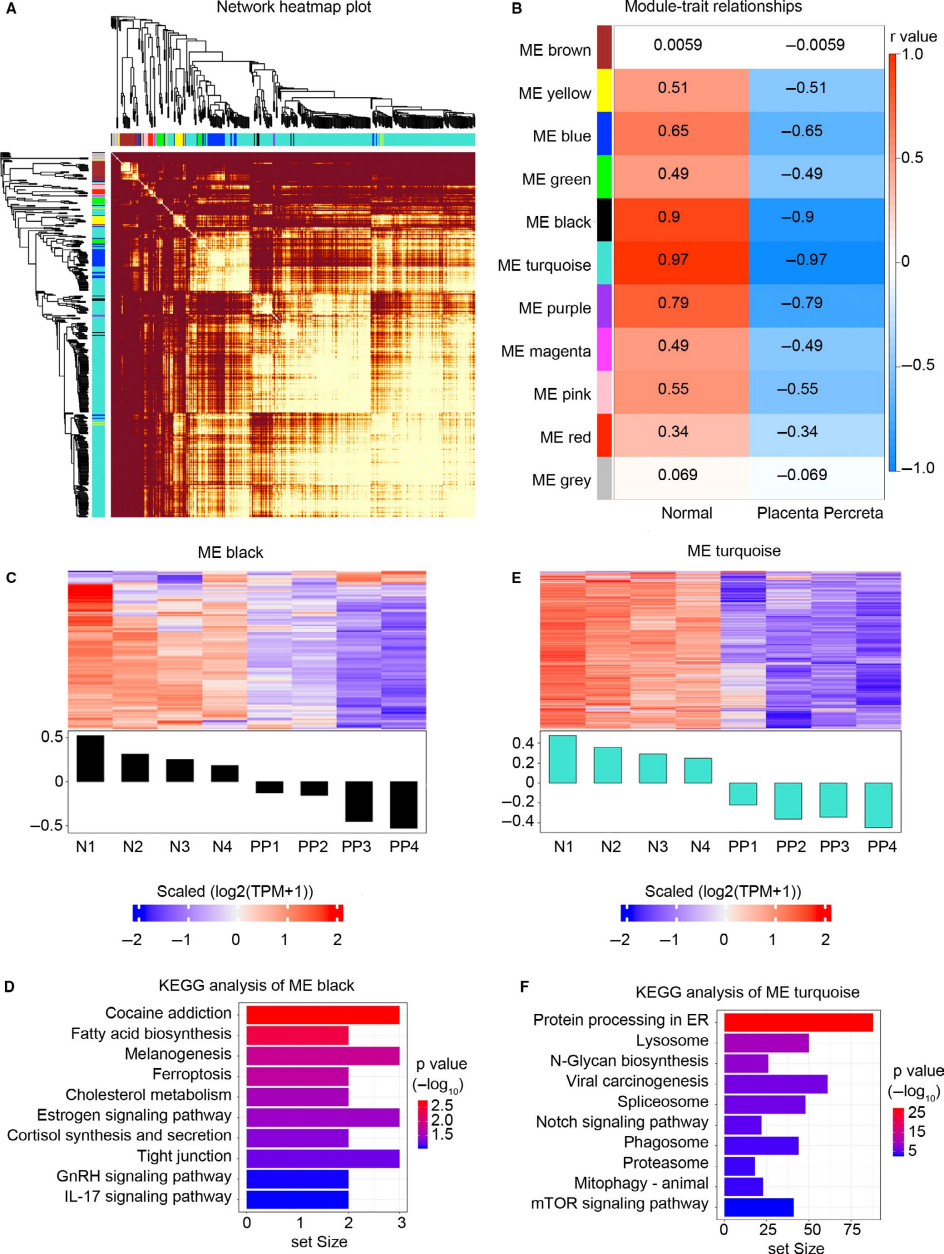
Weighted gene co‐expression network analysis of dysregulated protein‐coding genes. A, Network heatmap plot of dysregulated protein‐coding genes in placental tissue of the placenta percreta group. B, Specific co‐expression gene modules and their correlations. The red square indicates a positive correlation, the blue square indicates a negative correlation, and the white square indicates no correlation. The *r‐*value was included in each square. C, Heatmap shows the expression pattern of all genes in the ME black module across all 8 samples. D, KEGG analysis of dysregulated protein‐coding genes in the ME black module. The top 10 results are plotted. E, Heatmap shows the expression pattern of all genes in the ME turquoise module across all 8 samples. F, KEGG analysis of dysregulated protein‐coding genes in the ME turquoise module. The top 10 results are plotted

To select the cluster of hub coding genes involved in the pathogenesis of PP, a WGCNA was carried out to divide the deregulated coding genes into several clusters based on a similar expression trend. The network of all gene clusters is displayed as a heatmap (Figure 3A). The expression correlation was introduced as an important evaluation index to select functional modules. The ME black module and ME turquoise module were highly correlated with the pathogenesis of PP (Figure 3B). Coding genes in the ME black were down‐regulated and involved in cocaine addiction, fatty acid biosynthesis and melanogenesis (Figure 3C,D). Coding genes in the ME turquoise were also down‐regulated in PP and involved in protein processing in the endoplasmic reticulum (ER), lysosomes and N‐glycan biosynthesis (Figure 3E,F). These results indicated that coding genes in the ME black and ME turquoise play crucial roles in the pathogenesis of PP.

### 3.4 Down‐regulation of Wnt5A and MAPK13 in PP

**FIGURE 4:**
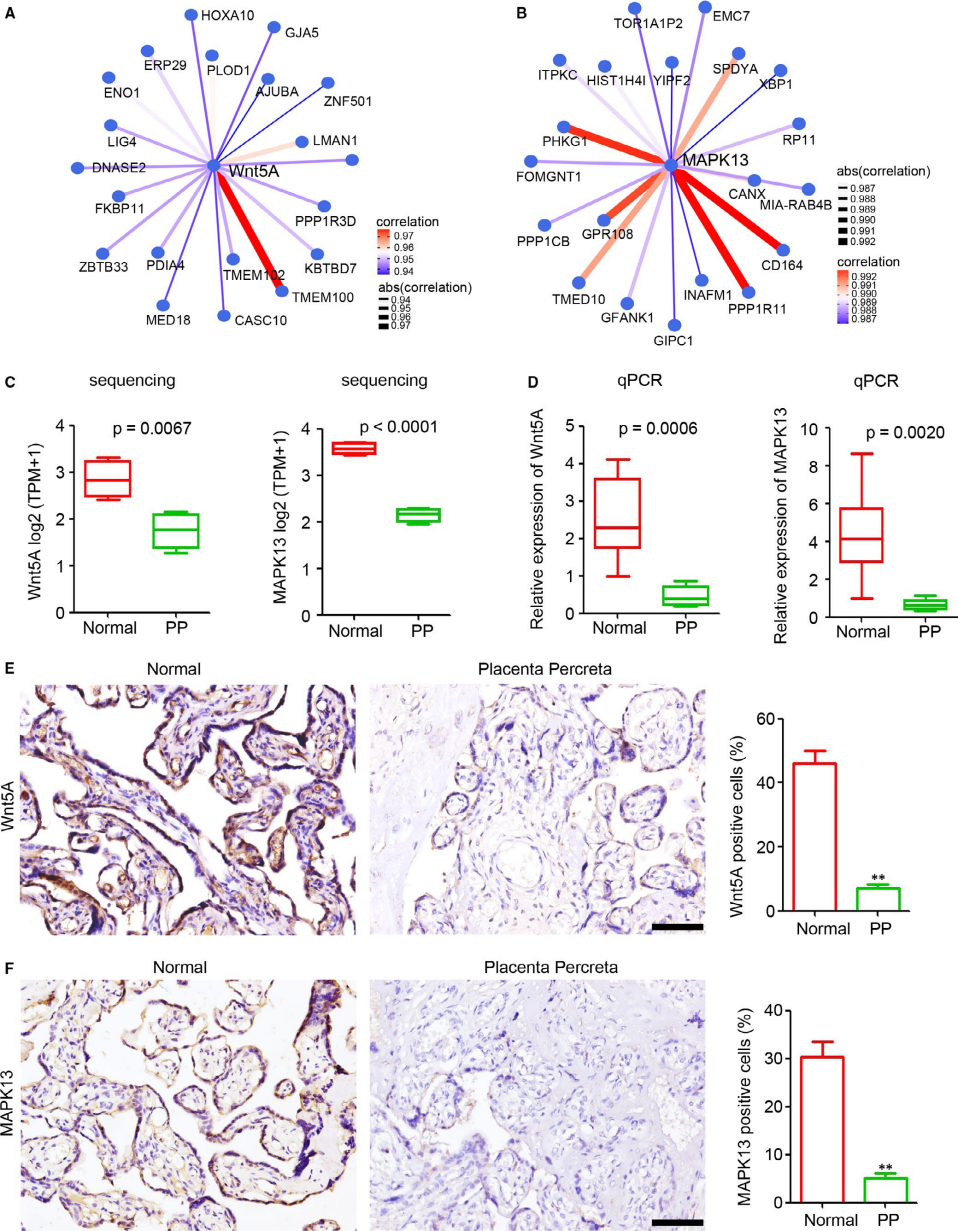
Down‐regulation of Wnt5A and MAPK13 in placenta percreta (PP). A, Analysis of the correlation between *Wnt5A* expression and the expression of other protein‐coding genes in normal and PP tissues. The top 20 protein‐coding genes are plotted. B, Analysis of the correlation between *MAPK13* expression and the expression of other protein‐coding genes in normal and PP tissues. The top 20 coding genes are plotted. C and D, *Wnt5A* and *MAPK13* expression levels in placental tissue of normal and PP groups, as determined by RNA sequencing (C) and qPCR (D, n = 6 per group; **P < 0.01 compared with the normal group. GAPDH was used as a loading control.). E and F, Immunohistochemical staining of Wnt5A and MAPK13 in placental tissue of normal and PP groups. The positive cells in each sample were analysed (n = 6 per group, **P < 0.01, compared with the normal group.). Scale bar = 100 μm

WGCNA analysis suggested that both of ME black and ME turquoise play crucial roles in the pathogenesis of PP. Correlation analysis among coding genes was performed in ME black and ME turquoise to identify the potential hub genes, which have tight correlation with other coding genes. Function prediction and article research suggested that Wnt5A and MAPK13 in ME turquoise are the hub genes involved in cell differentiation, cell proliferation, cell migration and placenta microenvironment.^31^, ^32^, ^33^ Thus, further analysis and experiments were performed based on the hub genes Wnt5A and MAKP13 in ME turquoise. Our results indicated that Wnt5A was a potential hub coding gene, which correlated with *TMEM100*, *ENO1*, *HOXA10* and *LMAN1* (Figure 4A), whereas the other potential hub coding gene, *MAPK13*, correlated with *CD164*, *PHKG1*, *SPDYA* and *INAFM1* (Figure 4B). Sequencing and qPCR indicated that the expression of *Wnt5A* and *MAPK13* was significantly down‐regulated in PP (Figure 4C,D). Furthermore, IHC staining was performed to detect Wnt5A and MAPK13 expression in the placental tissues of the normal and percreta groups. Fewer Wnt5A positive cells were observed in the PP group than in the normal group (Figure 4E). Similar results were determined for MAPK13 expression in the normal and PP groups (Figure 4F). Collectively, these results showed that down‐regulated Wnt5A and MAPK13 in the ME turquoise are potential hub coding genes involved in the pathogenesis of PP.

### 3.5 Down‐regulation of lncRNA PTCHD1‐AS and PAPPA‐AS1 in PP

**FIGURE 5:**
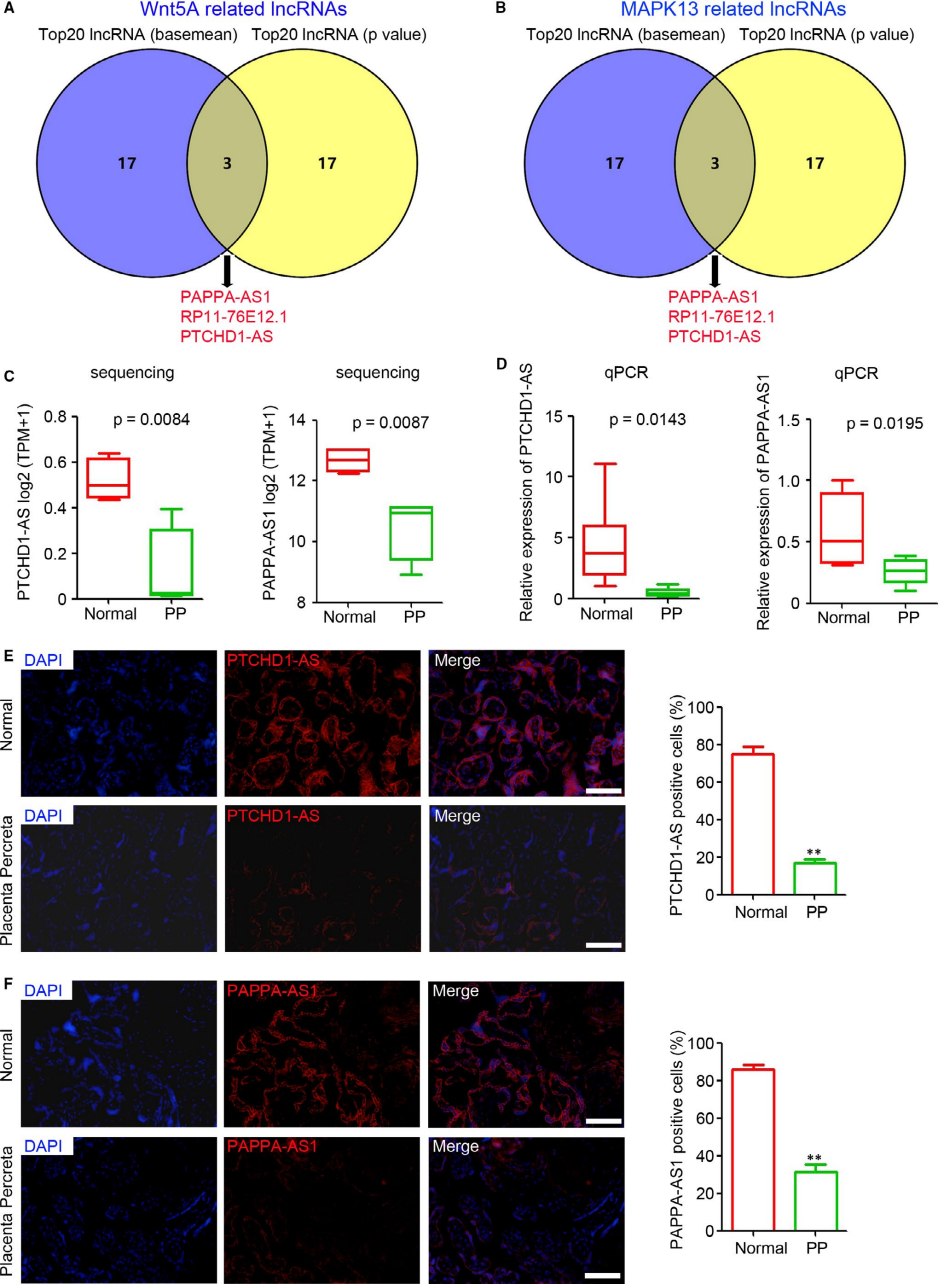
Down‐regulation of lncRNA *PTCHD1‐AS* and *PAPPA‐AS1* in placenta percreta (PP). Venn analysis of the top 20 (A) Wnt5A‐related lncRNAs and (B) MAPK13‐related lncRNAs, ranked by basemean and *P* value. (C and D) lncRNA *PTCHD1‐AS* and *PAPPA‐AS1* levels in placental tissue of normal and PP groups, as determined by RNA sequencing (C) and qPCR (D, n = 6 per group; **P < 0.01 compared with the normal group. GAPDH was used as a loading control). (E and F) FISH analysis of lncRNA *PTCHD1‐AS* and *PAPPA‐AS1* expression in placental tissue of normal and PP groups. The positive cells in each sample were analysed (n = 6 per group; **P < 0.01, compared with the normal group). Scale bar = 100 μm

Next, we aimed to identify potential hub lncRNAs that correlated with Wnt5A and MAPK13. All deregulated lncRNAs in PP that correlated with Wnt5A and MAPK13 at the cut‐off *r* value >0.5 were ranked according to baseMean expression and *P* value (expression analysis between normal and PP group). The top 20 lncRNAs (Tables [Link], [Link], [Link], [Link]), ranked by the *P* value and baseMean (the mean of the counts divided by the size factors for the counts for a given conditions, produced by DESeq or DESeq2), were selected for the Venn analysis (https://bioinfogp.cnb.csic.es/tools/venny/). PTCHD1‐AS, RP11‐6E12.1 and PAPPA‐AS1 were potential hub lncRNAs that tightly correlated with the expression of Wnt5A (Figure 5A). A similar analysis was performed to select lncRNAs correlated with MAPK13, and the results showed that PTCHD1‐AS. RP11‐6E12.1 and PAPPA‐AS1 were also tightly correlated with the expression of MAPK13 (Figure 5B). RP11‐6E12.1 is a predicted lncRNA without exact sequence information. Thus, we focused on PTCHD1‐AS and PAPPA‐AS1 in the further investigation. Sequencing and qPCR demonstrated the down‐regulation of PTCHD1‐AS and PAPPA‐AS1 in the PP group compared with the normal group (Figure 5C,D). FISH staining was carried out to determine the expression of PTCHD1‐AS and PAPPA‐AS1 in placental tissues. Fewer PTCHD1‐AS positive cells were observed in the placental tissues of the percreta group than in the placental tissues of the normal group (Figure 5E). The down‐regulation of PAPPA‐AS1 was also observed in PP tissues, as evidenced by FISH staining (Figure 5F). These results indicated that PTCHD1‐AS and PAPPA‐AS1 are potential hub lncRNAs involved in the pathogenesis of PP by regulating Wnt5A and MAPK13 expression.

### 3.6 LncRNA PTCHD1‐AS and PAPPA‐AS1 were associated with the expression of Wnt5A and MAPK13

**FIGURE 6:**
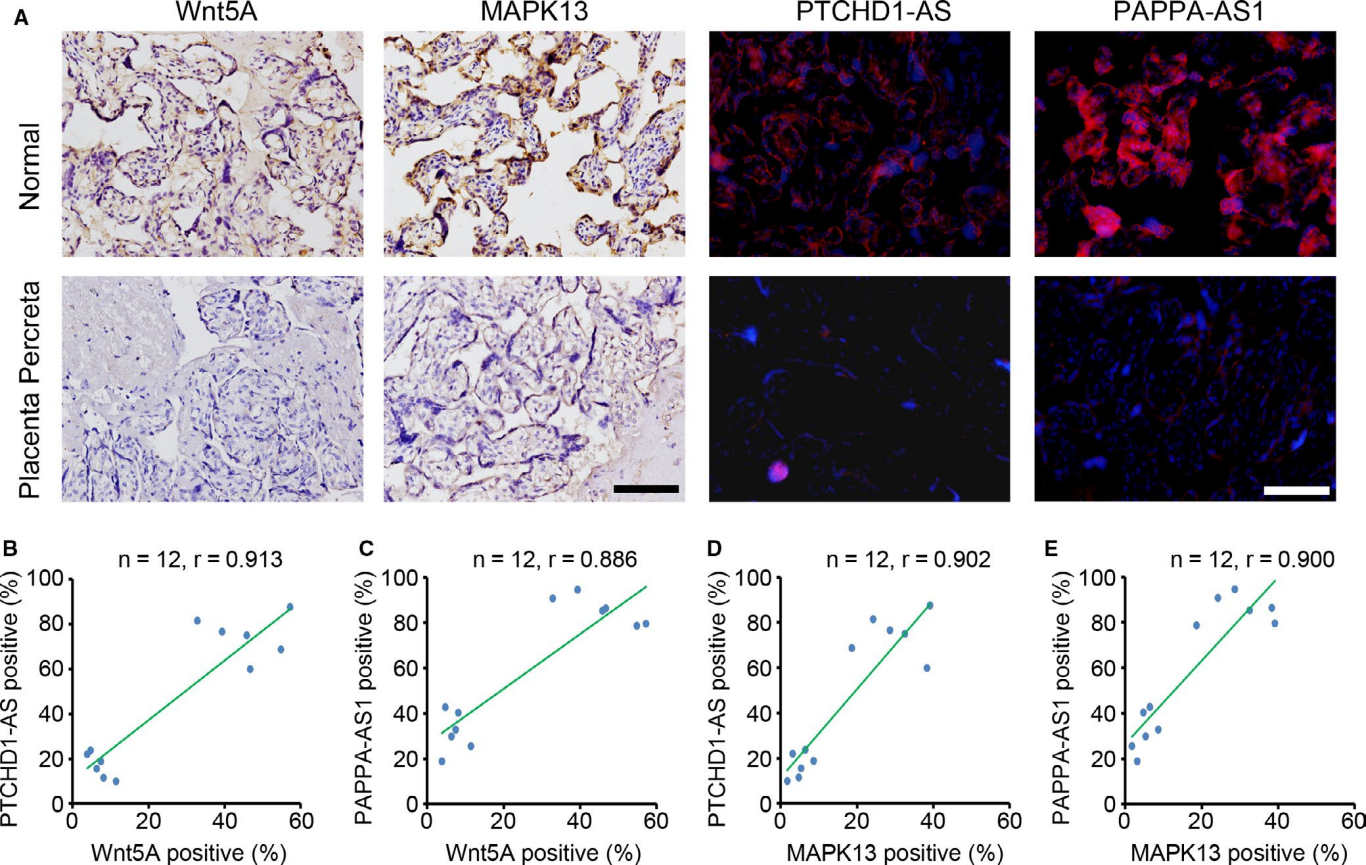
Expression levels of lncRNA *PTCHD1‐AS* and *PAPPA‐AS1* correlated with the expression levels of Wnt5A and MAPK13. A, The expression levels of Wnt5A, MAPK13, and lncRNA *PTCHD1‐AS*and *PAPPA‐AS1* in placental tissue of normal and placenta percreta (PP) groups. Scale bar = 100 μm. B‐E, The correlation between expression levels of Wnt5A, MAPK13, *PTCHD1‐AS* and *PAPPA‐AS1* in placental tissue of normal and PP groups (n = 6 in normal group and n = 6 in PP group). The *r‐*value is included in each square

Expression correlation analysis based on sequencing data indicated tight correlations among Wnt5A, MAPK13, PTCHD1‐AS and PAPPA‐AS1. Next, we tried to confirm the correlation based on IHC and FISH staining results in the normal and PP groups (Figure 6A). The expression of Wnt5A, MAPK13, TCHD1‐AS and PAPPA‐AS1 were recorded according to the average percentage of positive cells in each sample. Wnt5A expression was correlated with the expression of PTCHD1‐AS and PAPPA‐AS1 with *r* values of 0.913 and 0.886, respectively (Figure 6B,C). MAPK13 expression was correlated with the expression of PTCHD1‐AS and PAPPA‐AS1 with *r* values of 0.902 and 0.900, respectively (Figure 6D,E). Collectively, these results suggested that Wnt5A and MAPK13 expression is correlated with the expression of PTCHD1‐AS and PAPPA‐AS1.

### 3.7 LncRNA PTCHD1‐AS and PAPPA‐AS1 regulated the expression of Wnt5A and MAPK13 through miRNAs

**FIGURE 7:**
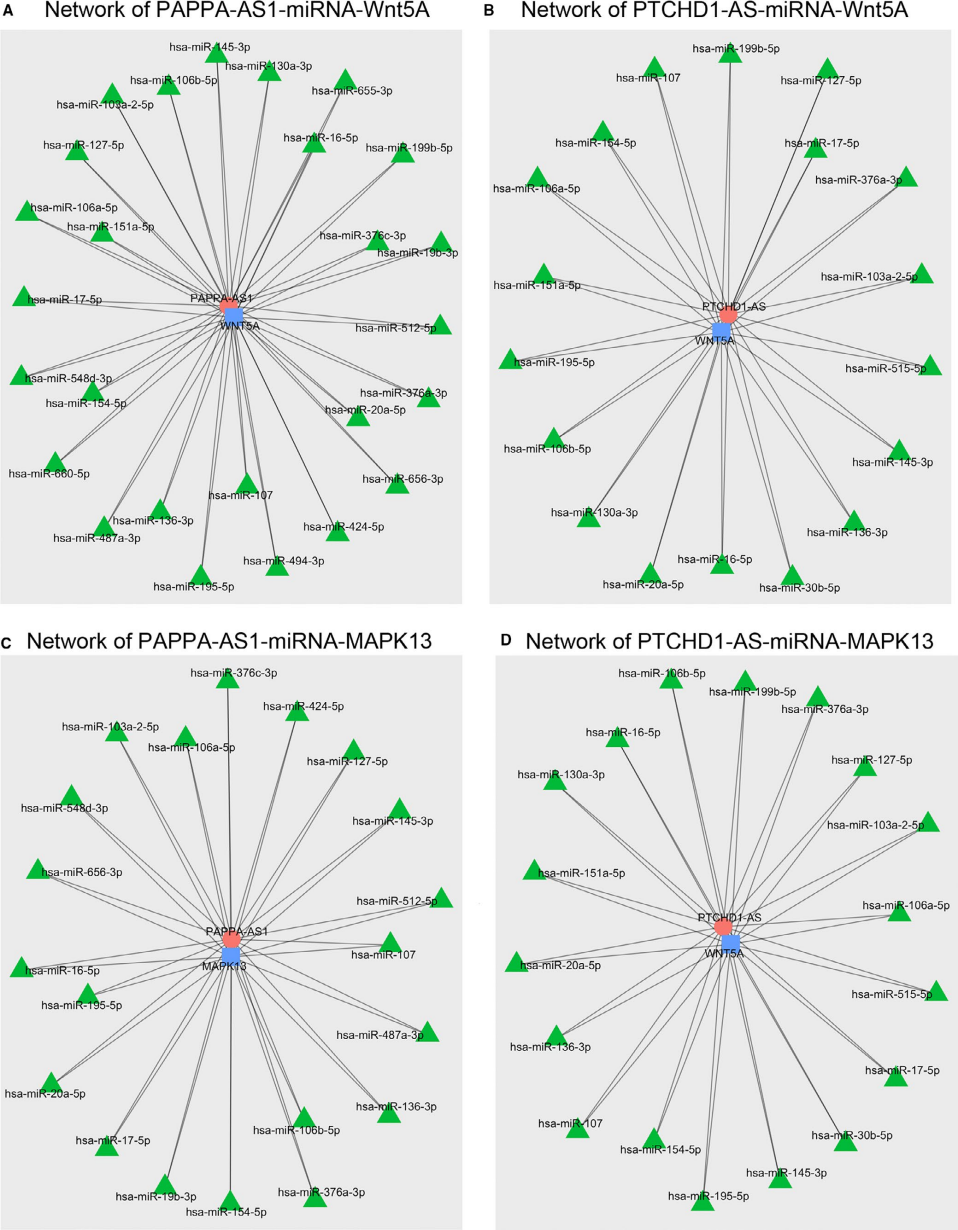
Network of lncRNAs, miRNAs and mRNAs. Expression connections of (A) *PAPPA‐AS1*‐miRNA‐Wnt5A, (B) *PTCHD1‐AS*‐miRNA‐Wnt5A, (C) *PAPPA‐AS1*‐miRNA‐MAPK13 and (D) *PTCHD1‐AS*‐miRNA‐MAPK13, based on target prediction. The top 20 miRNAs ranked by Pearson's correlation *r*‐values are displayed

Targeting miRNAs is an efficient and widely accepted method of lncRNA regulation to regulate the expression of coding genes. Thus, we aimed to construct a lncRNA‐miRNA‐mRNA network based on miRNA target prediction and the expression correlation determined by sequencing. PAPPA‐AS1 may regulate Wnt5A expression via interactions with miR‐127‐5p, ‐17‐5p, ‐145‐3p, and other 17 miRNAs (Figure 7A). PTCHD1‐AS may regulate Wnt5A expression via the formation of competing endogenous RNA with miR‐107, ‐154‐5p, ‐127‐5p and other 17 miRNAs (Figure 7B). Further analysis indicated that 20 miRNAs involved in MAPK13 expression are regulated by PAPPA‐AS1, including miR‐424‐5p, ‐107, and ‐17‐5p (Figure 7C). PTCHD1‐AS may also regulate MAPK13 expression via interactions with miR‐20a‐5p, ‐16‐5p, ‐17‐5p, and other 17 miRNAs (Figure 7D). These results were used to construct the lncRNA‐miRNA‐mRNA network.

## 4 DISCUSSION

In the present study, the results indicated that many lncRNAs, miRNAs and coding genes are deregulated in PP tissues compared with normal tissues. WGCNA analysis identified the potential hub coding genes in the ME turquoise, *Wnt5A* and *MAPK13*, were down‐regulated in PP tissues and correlated with the expression of lncRNAs PTCHD1‐AS and PAPPA‐AS1 via miRNA sponge network. Collectively, we demonstrated the expression profile of lncRNAs, miRNAs, and coding genes in PP and indicated that lncRNAs may play a crucial role in the pathogenesis of PP via interactions with miRNAs.

Placenta previa, previous caesarean delivery, endometrial ablation and other uterine surgeries are major risk factors for PAS.^1^ Clarifying the molecular mechanism would expand our understanding of the pathogenesis of PAS and provide potential predictive and therapeutic targets for PAS. Placenta accreta, placenta increta and PP are the three main categories of PAS, and PP is the most serious PAS.^1^ In our study, strict selecting criteria for the experimental group were as follows: (a) all the patients were diagnosed with pernicious placenta previa with PP; (b) all patients underwent hysterectomy; (c) no infection and no internal and surgical complications; (d) all cases were excluded from other pregnancy complications. Magnetic resonance imaging, ultrasonic examination, and H&E staining were employed to investigate the pathological features of the placental tissues used for sequencing analysis. Our results demonstrated that all of the samples in PP group get the typical features PP, which guarantees the uniformity of used samples. Thus, the uniformity of samples in per group decreased the heterogeneity in group and increased the credibility of sequencing results. The placental tissues of pregnant women with other surgical indications (scarred uterus, foetal macrosomia, breech presentation or cord around the neck) were collected as the normal group. According to the strict selection criteria, few samples were included in our study, which is the limitation of the present study.

In the present study, WGCNA was performed to select potential hub coding genes that were deregulated in the PP tissues. The ME turquoise that contained down‐regulated coding genes in PP tissues was used, and two potential hub coding genes, *Wnt5A* and *MAPK13*, were selected for further analysis based on the correlation expression analysis. Wnt5A plays a crucial role in placental growth and survival by increasing the proliferation of villous cytotrophoblasts and cell column trophoblasts.^31^ The deletion of *Wnt5A* often results in disorderly epithelial projections, crypt formation, embryo spacing, and impaired implantation, which suggests that Wnt5A is necessary for pregnancy success.^33^ The abnormal up‐regulation of *Wnt5A* accelerates the pathogenesis of preeclampsia via the regulation of trophoblast invasion,^34^ placental angiogenesis,^32^ decidualization^35^ and induction of the expression of ICAM‐1 and VCAM‐1 in trophoblast cells.^36^ In the present study, we demonstrated significant down‐regulation of *Wnt5A* in PP tissues, which indicated that Wnt5A may play a crucial role in the pathogenesis of PAS. Thus, we speculated that a moderate expression of Wnt5A is required for pregnancy success and normal parturition. Based on the similarities in the WGCNA analysis between our study and previous studies^32^, ^33^, ^34^, ^35^, ^37^, ^38^ on *Wnt5A* expression and the potential functions, we have reason to believe that the coding genes in the ME turquoise, including *MAPK13*, which was also down‐regulated in PP tissues, are crucial regulators in the pathogenetic process of PAS.

A previous study by Wu et al reported 329 deregulated lncRNAs in five tissue specimens of placental implantation tissue compared with the paired adjacent normal placental tissues.^39^ In our study, to better distinguish the tissues and clarify the heterogeneity among pregnant women, the placental tissues of pregnant women with PAS and normal pregnant women with operation indications were collected for sequencing. In total, 541 lncRNAs were deregulated in the PP tissues compared with the normal placenta group. After selecting the hub coding genes by WGCNA analysis, the correlation between lncRNAs and hub coding genes was established based on the expression in the eight sequenced samples. Two potential hub lncRNAs, PTCHD1‐AS and PAPPA‐AS1, which positively correlated with Wnt5A and MAPK13 expression, were selected. PTCHD1‐AS deletions are risk factors for autism spectrum disorder^40^ and the copy number of PTCHD1‐AS is involved in neurodevelopmental disorders.^41^ PAPPA‐AS1 is an antisense lncRNA of PAPPA, which is a key regulator of insulin‐like growth factor bioactivity and an important indicator for pregnancy and gestation.^42^, ^43^, ^44^, ^45^ Currently, the expression and potential function of PTCHD1‐AS and PAPPA‐AS1 in PAS are unclear. However, our study suggested that both PTCHD1‐AS and PAPPA‐AS1 are down‐regulated in PP tissues and correlate with the expression of Wnt5A and MAPK13, as evidenced by sequencing data and histological data. Thus, we speculated that deregulated lncRNAs may play a crucial role in the pathogenesis of PAS. However, further investigations are needed to determine its functions.

Previous studies have demonstrated that miR‐125a, ‐34a and ‐29a/b/c are involved in the pathogenesis of PAS.^18^, ^46^, ^47^ In our study, 382 miRNAs, including miR‐125a, ‐34a, and ‐29a/b/c, were deregulated in PP tissues compared with normal placental tissues. Based on the miRNA target prediction and expression correlation analysis, we constructed the lncRNA‐miRNA‐mRNA network. Several miRNAs, such as miR‐17‐5p, ‐127‐5p and ‐107, which are involved in cell proliferation, apoptosis and angiogenesis,^48^, ^49^, ^50^, ^51^, ^52^, ^53^ mediate the regulation among lncRNAs PTCHD1‐AS and PAPPA‐AS1 and Wnt5a and MAPK13. The construction of the lncRNA‐miRNA‐mRNA network provided the potential underlying mechanism of lncRNA in the regulation of the pathogenesis of PAS, but further experiments are needed to confirm this finding.

Collectively, the present study demonstrated the crucial role of lncRNAs in the pathogenesis of PAS, which may provide a predictive biomarker and therapeutic target for PAS. However, further investigations are needed to expand the sample size and clarify the molecular mechanism involved.

## CONFLICTS OF INTEREST

All authors declare that there are no conflicts of interest.

## AUTHOR CONTRIBUTIONS

**Qingyuan Jiang:** Formal analysis (equal); investigation (equal); project administration (equal); software (equal); writing – original draft (equal). **Lei Dai:** Investigation (equal); writing – original draft (equal). **Na Chen:** Investigation (supporting); methodology (supporting). **Junshu Li:** Investigation (supporting); methodology (supporting). **Yan Gao:** Investigation (supporting). **Jing Zhao:** Data curation (supporting); investigation (supporting); methodology (supporting); resources (supporting). **Li Ding:** Data curation (supporting); methodology (supporting); resources (supporting). **Chengbin Xie:** Investigation (supporting); methodology (supporting). **Xiaolian Yi:** Investigation (supporting); methodology (supporting). **Hongxin Deng:** Conceptualization (equal); funding acquisition (equal); project administration (equal); supervision (equal). **Xiaodong Wang:** Conceptualization (equal); funding acquisition (equal).

## Supporting information

Fig S1

Table S1

Table S2

Table S3

Table S4

## Data Availability

All data generated and/or analysed during this study are included in this published article.
