# Supplementary figures and images for: Integrative analysis provides multi‐omics evidence for the pathogenesis of placenta percreta
Source: J Cell Mol Med. 2020 Oct 21;24(23):13837–52. doi: 10.1111/jcmm.15973 (PMC7754008; doi:10.1111/jcmm.15973)

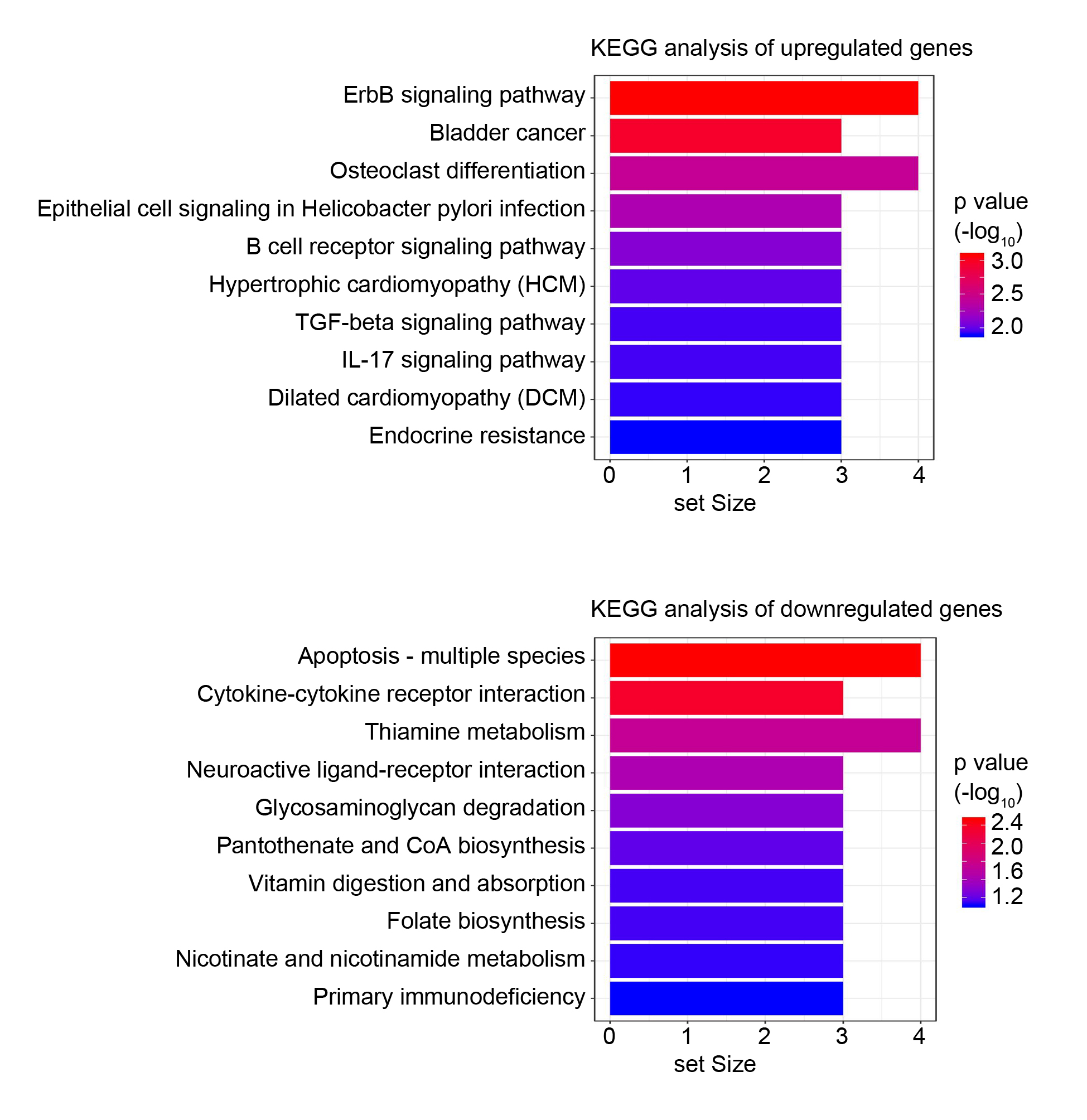

Supplement: Supplementary file 1 — Fig S1 [file JCMM-24-13837-s001.jpg]
