# Supplementary material for: Integrative analysis provides multi‐omics evidence for the pathogenesis of placenta percreta
Source: J Cell Mol Med. 2020 Oct 21;24(23):13837–52. doi: 10.1111/jcmm.15973 (PMC7754008; doi:10.1111/jcmm.15973)
Supplement: Supplementary file 2 — Table S1 [file JCMM-24-13837-s002.docx]

**Supplementary Table 1 Top 20 lncRNAs correlated with Wnt5A expression ranked by P value (fold change)**

| **lncRNA** | **P value** | **Basemean (counts)** |
| --- | --- | --- |
| \| RP11-513G11.3 \| \| --- \| \| RP11-20F18.1 \| \| RP11-76E12.1 \| \| PTCHD1-AS \| \| RP11-520D19.2 \| \| GS1-433O24.1 \| \| RP11-513G11.4 \| \| RP11-494I9.2 \| \| RP11-1038A11.3 \| \| RP11-655G22.1 \| \| CTD-2008P7.8 \| \| XX-CR54.1 \| \| MYO16-AS1 \| \| RP11-96K19.2 \| \| PAPPA-AS1 \| \| SLC6A1-AS1 \| \| LINC00370 \| \| AC003986.6 \| \| RP11-693J15.6 \| \| RP11-333A23.4 \| | \| 4.80E-07 \| \| --- \| \| 2.03E-06 \| \| 2.95E-05 \| \| 4.19E-05 \| \| 7.00E-05 \| \| 0.000124204 \| \| 0.000207102 \| \| 0.000228275 \| \| 0.000329827 \| \| 0.000396728 \| \| 0.000461562 \| \| 0.000493576 \| \| 0.00051476 \| \| 0.000515462 \| \| 0.000602444 \| \| 0.000775395 \| \| 0.00085415 \| \| 0.000903074 \| \| 0.000952441 \| \| 0.001106984 \| | \| \| 805.3359034 \| \| --- \| \| 6.767521677 \| \| 9051.76317 \| \| 5247.142907 \| \| 291.9072069 \| \| 557.8515849 \| \| 453.3443772 \| \| 149.3271058 \| \| 764.4180583 \| \| 23.11396619 \| \| 389.6658119 \| \| 834.5080851 \| \| 87.3540501 \| \| 1833.990701 \| \| 220297.5719 \| \| 47.63532805 \| \| 38.14451537 \| \| 142.9198096 \| \| 25.76678143 \| \| 803.5227478 \| \| \| --- \| --- \| --- \| --- \| --- \| --- \| --- \| --- \| --- \| --- \| --- \| --- \| --- \| --- \| --- \| --- \| --- \| --- \| --- \| --- \| --- \| |
