# Supplementary material for: Integrative analysis provides multi‐omics evidence for the pathogenesis of placenta percreta
Source: J Cell Mol Med. 2020 Oct 21;24(23):13837–52. doi: 10.1111/jcmm.15973 (PMC7754008; doi:10.1111/jcmm.15973)
Supplement: Supplementary file 3 — Table S2 [file JCMM-24-13837-s003.docx]

**Supplementary Table 2 Top 20 lncRNAs correlated with Wnt5A expression ranked by basemean**

| **lncRNA** | **P value** | **Basemean (counts)** |
| --- | --- | --- |
| \| PAPPA-AS1 \| \| --- \| \| AC068134.8 \| \| RP11-323N12.5 \| \| AC003991.3 \| \| RP11-400F19.6 \| \| CTC-458I2.2 \| \| ATP1A1-AS1 \| \| RP1-197B17.3 \| \| RP11-76E12.1 \| \| TINCR \| \| LINC00474 \| \| LINC00967 \| \| RP11-1070N10.6 \| \| PTCHD1-AS \| \| RP1-43E13.2 \| \| AC005523.2 \| \| RP11-670E13.5 \| \| RP11-78I14.1 \| \| RP11-1007I13.4 \| \| AC003075.4 \| | \| 0.000602444 \| \| --- \| \| 0.01102037 \| \| 0.009587291 \| \| 0.044442453 \| \| 0.043617471 \| \| 0.024807742 \| \| 0.006316814 \| \| 0.043128146 \| \| 2.95E-05 \| \| 0.003158867 \| \| 0.039110905 \| \| 0.006988615 \| \| 0.011380683 \| \| 4.19E-05 \| \| 0.027585587 \| \| 0.02106367 \| \| 0.003763982 \| \| 0.00910857 \| \| 0.035639507 \| \| 0.043013464 \| | \| 220297.5719 \| \| --- \| \| 62010.5503 \| \| 26078.86044 \| \| 21843.8955 \| \| 21748.25922 \| \| 13660.48573 \| \| 11469.32808 \| \| 9655.647218 \| \| 9051.76317 \| \| 8949.518269 \| \| 6518.312306 \| \| 5620.17972 \| \| 5282.674132 \| \| 5247.142907 \| \| 5142.92673 \| \| 4780.298827 \| \| 3796.757306 \| \| 3318.638206 \| \| 3232.549386 \| \| 3045.350867 \| |
